# Connected health in cancer survivorship: Evaluating the usability and utility of the cancer thriving and surviving programme in Ireland

**DOI:** 10.1007/s11845-025-03931-6

**Published:** 2025-03-21

**Authors:** Isaiah Gitonga, Deirdre Desmond, Louise Mullen, Dorothy Thomas, Cathleen Osborne, Bernie O’Loughlin, Rebecca Maguire

**Affiliations:** 1 Department of Psychology, Maynooth University, Maynooth, Ireland; 2 Assisting Living and Learning Institute, Maynooth University, Maynooth, Ireland; 3 Health Service Executive, National Cancer Control Programme, Dublin, Ireland

**Keywords:** Cancer survivorship, Cancer thriving and surviving programme, Connected health, Ireland, Telehealth, Usability

## Abstract

**Background:**

Cancer survivorship care has become increasingly complex, with a growing population of people living with and beyond the disease requiring holistic support and follow-up. Connected health (CH) offer a promising solution to enhance care delivery.

**Aim:**

This study evaluated the usability and effectiveness of CH, and motivations of participants in the Cancer Thriving and Surviving (CTS) programme in Ireland.

**Methods:**

A cross-sectional survey of persons living with and beyond cancer (PLWBC) who completed the CH-delivered CTS was conducted between December 2022 and April 2023. Closed and open-ended questions captured participants experiences and motivations. Telehealth Usability Questionnaire (TUQ) assessed the CH usability. Qualitative content analysis examined recurring themes in participant responses.

**Results:**

Forty-four participants who engaged in CTS completed the survey. Participants were predominantly female (88%), diagnosed with breast cancer (76%), and had third-level education or higher (86%). Slightly over one third (36%) were in full time employment. Motivations for engaging in CTS included seeking peer support, psychosocial assistance, and practical self-management tools. Most respondents agreed that the programme improved their psychological wellbeing (90%), quality of life (76%) and helped them take more control of their health (83%). TUQ scores indicated high usability of the CH systems.

**Conclusion:**

Findings suggest that the CH-delivered CTS programme effectively benefits PLWBC, improving psychological well-being and quality of life. The high CH system usability and positive user experiences highlight its potential to complement in-person care, supporting the continued development and evaluation of CH systems to enhance cancer survivorship, particularly within Irish digital health initiatives.

## Introduction

As both incidence and survival rates of cancer grow [1], there is a corresponding increase in demand for healthcare services designed to assist those living with and beyond the disease [2, 3]. Healthcare systems are increasingly turning to technology, driven by the need to reduce costs while expanding access to services [4, 5]. One area of recent technological advancement is Connected Health (CH), a sociotechnical approach to healthcare that links people, processes, and technology [6, 7]. CH is an overarching term encompassing e-health, wearables, sensor technology, and mhealth, among other elements [8, 9]. CH holds great potential for supporting people impacted by long-term diseases through increased access to services, personalized care, and self-management [7, 10]. In the recent past, and particularly in the aftermath of the COVID-19 pandemic, there has been a rapid proliferation of CH technologies [11, 12]. While evidence for the benefits of these technologies continues to accumulate, their full potential is yet to be examined and exploited [13]. In cancer survivorship care, for instance, CH uptake remains unequal across different demographic and socioeconomic groups[14], while the need for evaluation of CH use, efficacy, efficiency and sustainability remains.

In order to reap potential benefits of CH technologies, the delivery system has to be usable for both patients and clinicians [15]. Usability is the extent to which a product can be used to achieve specified goals with effectiveness, efficiency, and satisfaction in a specified context of use [16, 17]. Key components of usability are usefulness, ease of use, learnability, interface quality, interaction quality, reliability, and user satisfaction [18]. While early work in CH usability evaluation was primarily focused on user satisfaction [19, 20], recent work incorporated usefulness, ease of use, and interaction quality [21, 22], reflecting the rapidly changing technological landscape, and pointing to a need for continuous evaluation.

While the benefits of CH are evident, understanding patients’ motivations to engage in CH delivered interventions is crucial [23]*.* A significant body of literature underscores how key motivators include the convenience offered by CH [24], need for social and peer connection [25] and improved access to care and support [26], particularly for those with mobility challenges. Additionally, CH can provide personalized content, self-management tools, and educational materials that empower PLWBC to actively participate in their care [14, 27]. Patient characteristics play a significant role in shaping individual preferences and influencing engagement patterns [14, 28]. For example age and comfort with technology are important considerations [29]. Socioeconomic factors, including income, education, and health literacy, can also impact access to technology and digital literacy skills, highlighting the need for targeted interventions to ensure equitable access [14].

In recent years, Ireland has made strides in developing its eHealth infrastructure. For instance, the eHealth Ireland strategy and the digital framework 2024–2030 [39] outlines a vision for a CH-enabled health service, with a focus on improving access to care, empowering patients, and enhancing efficiency [30]. In this light, several survivorship programmes have since been established, delivered by the government [31] or charitable organisations such as the Irish Cancer Society [32]. One such programme is the Cancer Thriving and Surviving (CTS) programme. To enhance reach, the majority of these programmes are delivered both in person and virtually via CH systems.

## The cancer thriving and surviving programme

The CTS programme is the first nationwide survivorship initiative implemented by the National Cancer Control Programme (NCCP) in response to the National Cancer Strategy 2017–2026 [33] recommendation for the NCCP to work with organisations to develop and implement survivorship programmes. CTS is an evidence-based, self-management programme designed to empower cancer patients transitioning from active treatment to survivorship. Adapted from the Stanford Chronic Disease Self-Management Programme [34, 35], the CTS focuses on rebuilding self-confidence, adjusting to changed self-image, developing self-management skills, and promoting overall well-being. The programme was originally developed by Macmillan Cancer Support in the UK [36], and the Stanford Patient Education Research Centre [37] and has since been positively evaluated for feasibility and acceptability in the UK [36], Ireland [38] and the USA [39].

Initially delivered in-person, the CTS programme transitioned to an online format in response to the COVID-19 pandemic and the closure of in-person centres. A pilot and subsequent successful roll out demonstrated preliminary efficacy. Since then, the programme is now offered via both CH and in person, in over 20 acute hospital and community centres nationwide. As of 2023, more than 600 PLWBC had participated in the programme [31]. The programme involves six sessions each conducted over 2.5 hrs per week, for six weeks. Sessions are facilitated by two trained leaders, at least one of whom is a PLWBC. The programme accommodates 12–16 participants and covers topics such as self-management, well-being, cancer prevention, long-term treatment effects, and psychosocial support. For CH delivery, participants require stable internet access and compatible devices like smartphones, tablets, or computers to access the programme via Zoom, a videoconferencing platform [40]. While more people impacted by cancer continue to benefit from the programme across the delivery modalities, the utility and usability of the CH-delivered CTS has not been evaluated. The study sought to understand the usability of CH systems in delivery of CTS, its utility in supporting wellbeing and quality of life (QoL) of PLWBC, and motivations to complete the programme via this modality.

## Methods

### Study design

A cross-sectional survey design was adopted, targeting patients who had completed the CH-delivered CTS programme. The questionnaire combined a mixture of closed and open-ended questions to capture a comprehensive view of participant experiences. Specifically, questions asked about participant motivations for engaging in the CTS programme, the supports received, and the perceived usability in supporting psychological wellbeing and QoL. Ethical approval for this study was granted by Maynooth University Social Research Ethics subcommittee (Number SRESC-2022–2475301).

### Participants

Patients who had completed primary cancer treatment were invited to participate in the study. Eligibility criteria included: (i) having participated in the CH-delivered CTS programme, and (ii) being aged 18 years or older. It did not matter if they were in remission, stable disease or progressive disease.

### Recruitment strategy

Participants were recruited between December 2022 and April 2023. Recruitment was conducted by circulating an invitation to participate through the NCCP’s newsletters and cancer support centre networks, and by sharing the study details on social media platforms. Eligible participants provided consent and completed the survey questionnaire hosted on the Qualtrics platform [41].

### Instruments

#### Sociodemographic and Health details

Demographic characteristics (age, gender, education level, employment status, and urban or rural residence), type of cancer, time since diagnosis and completion of primary treatment, and treatments received were recorded.

#### Telehealth Usability Questionnaire 

The Telehealth Usability Questionnaire (TUQ) [18] was used to assess the usability of CH systems; respondents rated each question on a 7-point Likert scale (1: strongly disagree to 7: strongly agree). The TUQ is a measurement tool with good psychometric properties [42]. TUQ has been widely used to measure telehealth usability among various patient groups, including within an Irish population [43]. The higher the overall average score, the higher the usability of the telehealth system.

#### Motivations, supports and satisfaction 

To gather in-depth responses on participants' motivations, supports received, and the most useful elements of the programme, open-ended questions were posed. Specifically, the following questions were included: 

1. What was your main motivation for participating in the online programme?
2. What support did you receive to enable you to complete the online programme?
3. What components of the online programme did you find most useful as pertaining to your psychological well-being?

Additionally, participants were asked to rate their agreement with statements using a 5-point Likert scale regarding the impact of the programme on their psychological well-being, QoL and empowerment. One such statement was* ‘participation in the CH delivered CTS programme helped improve my psychological wellbeing.’*

### Data analysis

Descriptive statistics, including mean scores and standard deviations (SD), were calculated for continuous measures, while frequencies and percentages were calculated for categorical measures. To examine differences in TUQ scores across sociodemographic and disease categories, independent t-tests and ANOVA were used for normally distributed data. For non-normally distributed data, non-parametric tests, including the Mann–Whitney U and Kruskal–Wallis tests, were employed [44]. Statistical significance was set at *p* < 0.05. Open text responses were analysed through qualitative content analysis [45]. This method involves identifying, analysing, and reporting patterns (themes) within the data. As responses were often brief, the focus was on identifying and categorizing recurring themes and subthemes in the data [46]. After the primary researcher completed the initial coding and categorization of the data, the codes and the overall analysis were discussed with the rest of the research team to ensure that they accurately reflected participants' responses. Considering the brevity of the responses, qualitative content analysis allowed for a structured approach to interpret the data, both qualitatively in terms of the categories but also quantifying those responses by reporting the frequency of the code mentions. QDA Miner Lite [47], a free qualitative analysis software, was used.

## Results

### Sociodemographic characteristics and cancer history

Participants were predominantly female (88%, *n* = 38). Nearly three quarters (77%, *n* = 34) had breast cancer. Other diagnoses included Hodgkin's Lymphoma (*n* = 2), ovarian (*n* = 2), cervical (*n* = 2), prostate (*n* = 1), skin (*n* = 1), Ewing’s sarcoma (*n* = 1), and thyroid cancer (*n* = 1). A majority (86%, *n* = 38) had third level education and above. One third (36%, *n* = 16) were in full time work, with others either retired, on sick leave, or had not returned to work after cancer. Concerning cancer history, approximately three quarters (78%, *n* = 31) were diagnosed with cancer 2–5 years prior and slightly more than half (57% *n* = 20) had completed primary treatment within the last two years. Table 1 summarises the demographic and disease history characteristics of the sample.

**Table 1:** Sociodemographic characteristics and cancer history

Variable	Category	Frequency (*N* = 44)	Valid Percentage (%)
Age in years	29–44 Years	11	44
	45 + Years	14	56
	Non-Response	19	
Sex	Male	5	12
	Female	38	88
	Non-Response	1	
Education level	Post Secondary training and below	6	14
	Third Level and Above	38	86
Employment status	Working full time	16	36
	Others	28	64
Residence	Urban	23	54
	Rural	20	46
	Non-Response	1	
Time since diagnosis	Less than 2 Years	4	10
	2–5 years	31	78
	6 and above Years	5	12
	Non-Response	4	
Time since completing primary treatment	Less than 2 Years	20	57
	2–5 years	11	31
	6 and above Years	4	12
	Non-Response	9	

### CTS sessions

**Fig. 1:**
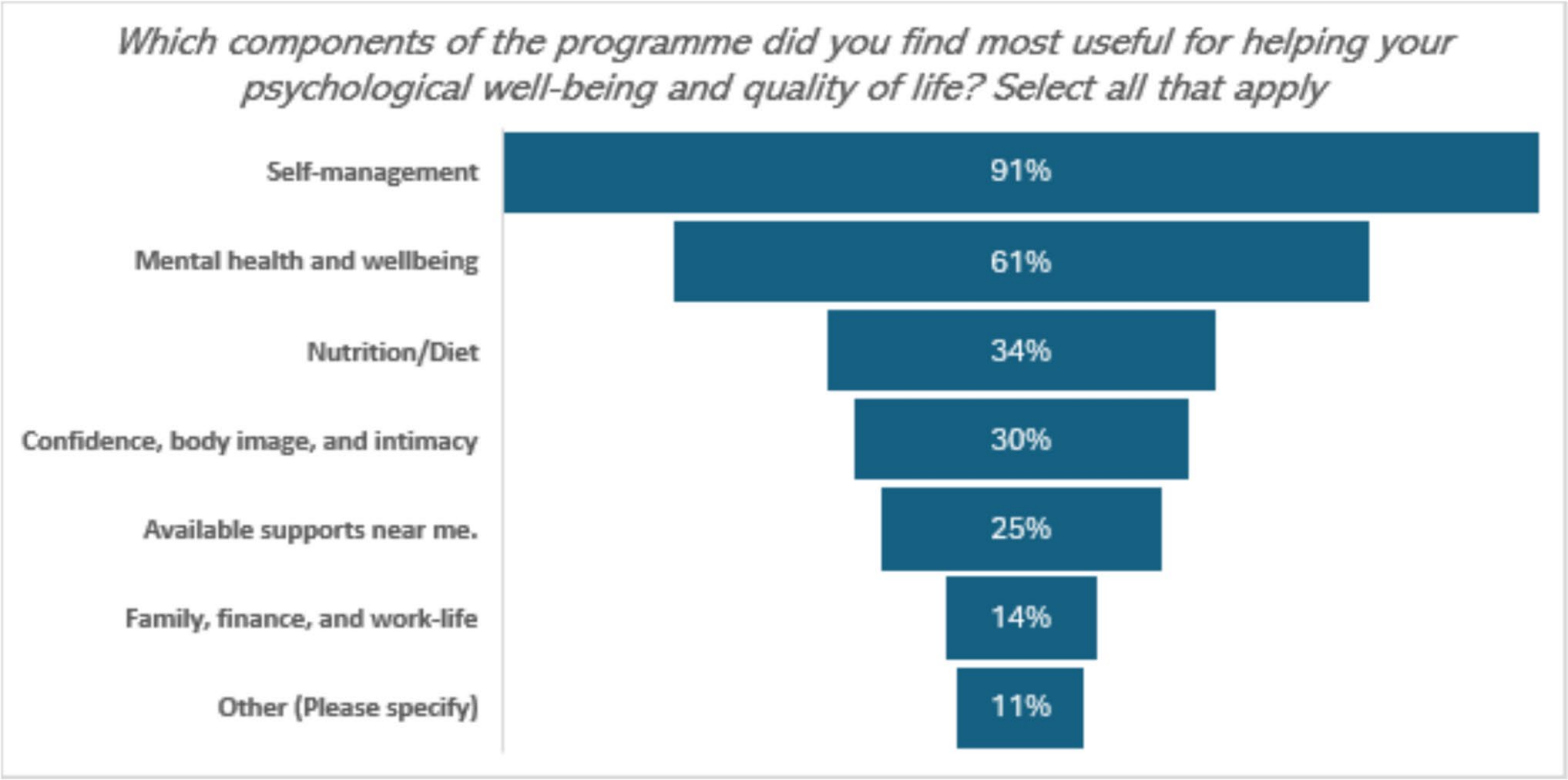
Usefulness of CTS sessions. *Others: Peer support, social aspect, meeting others

Nearly all participants had completed the required CTS sessions, with 43 out of 44 (98%) completing the prescribed six, 2.5-hr workshops between 2021 and 2022. One participant had completed five of the six sessions. Participants were asked to select the programme components that they found most useful in the programme, with the option to select all that applied. Most respondents endorsed self-management as the most useful aspect while family, finance, and work-life were least endorsed as shown in Fig. 1.

### Overall impact of participation

**Fig. 2:**
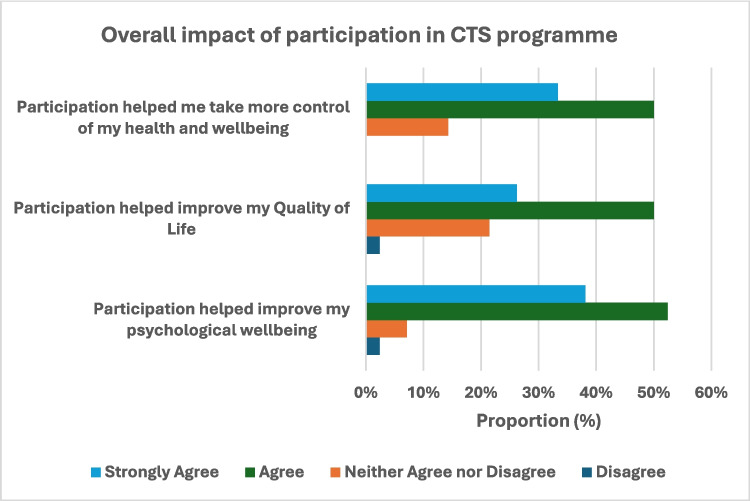
Overall impact of participation in CTS programme

Overall, the majority of participants agreed that engaging in the programme helped improve their psychological well-being (90%) and QoL (76%), and also that this allowed them to take more control of their health (83%) as shown in Fig. 2.

### Telehealth usability

Participants found the technology they used to access the programme useful (M = 4.58, SD = 1.78) and easy to use (M = 5.74, SD = 1.35). It was perceived as effective (M = 5.43, SD = 1.31) and reliable (M = 4.40, SD = 1.33). Overall satisfaction with technology used was high (M = 5.26, SD = 1.48). The total average score for CH usability was 5.18 (SD = 1.25), indicating a generally positive experience among the users. Table 2 shows the scores for each item, domains and the total average score.

**Table 2:** Telehealth Usability

Items (Numbers and Answers)	*N*	Mean ± SD	Range [1–7]
1. Telehealth improved my access to healthcare services	35	4.66 ± 1.91	[1–7]
2. Telehealth saved me time traveling to a hospital or specialist clinic	35	4.66 ± 2.17	[1–7]
3. Telehealth provided for my healthcare need	31	4.48 ± 1.96	[1–7]
Usefulness scale summary (Items 1–3)	**35**	**4.58 ± 1.78**	[1–7]
4. It was simple to use this system	31	6.00 ± 1.24	[1–7]
5. It was easy to learn to use the system	29	6.03 ± 1.12	[1–7]
6. I believe I could become productive quickly using this system	29	5.48 ± 1.64	[1–7]
7. The way I interacted with this system is pleasant	31	5.58 ± 1.36	[1–7]
8. I liked using the system	29	5.34 ± 1.65	[1–7]
9. The system is simple and easy to understand	30	6.03 ± 1.10	[1–7]
10. This system is able to do everything I would want it to be able to do	29	5.07 ± 1.77	[1–7]
Ease of use scale summary (Items 4–9)	**35**	**5.74 ± 1.357**	[1–7]
11. I can easily talk to the facilitator using the telehealth system	31	5.74 ± 1.34	[1–7]
12. I can hear the clinician clearly using the telehealth system	29	6.00 ± 1.16	[1–7]
13. I felt I was able to express myself effectively	32	5.53 ± 1.54	[1–7]
14. Using the telehealth system, I can see the facilitator as well as if we met in person	30	4.73 ± 1.95	[1–7]
15. I think the visits provided over the telehealth system are the same as in-person visits	33	3.79 ± 1.96	[1–7]
Effectiveness scale summary (Items 10–14)	**35**	**5.43 ± 1.31**	[1–7]
16. Whenever I made a mistake using the system, I could recover easily and quickly	32	5.44 ± 1.37	[1–7]
17. The system gave error messages that clearly told me how to fix problems	34	4.09 ± 1.58	[1–7]
18. I feel comfortable communicating with the facilitator using the telehealth system	31	5.68 ± 1.49	[1–7]
Reliability scale summary (Items 15–17)	**35**	**4.40 ± 1.33**	**[1–6.7]**
19. Telehealth is an acceptable way to receive healthcare services	34	4.53 ± 1.83	[1–7]
20. I would use telehealth services again	32	5.72 ± 1.44	[1–7]
21. Overall, I am satisfied with this telehealth system	35	5.46 ± 1.63	[1–7]
Satisfaction scale summary (Items 18–21)	**35**	**5.26 ± 1.48**	[1–7]
Total average score	**35**	**5.18 ± 1.25**	**[1–6.9]**

Likert scale used: 1: strongly disagree; 2: disagree; 3: somewhat disagree; 4: neutral; 5: somewhat agree; 6: agree; 7: strongly agree

### Sociodemographic characteristics and CH usability

There were no statistically significant differences in CH usability across age, sex, education level, employment status, residence, time since diagnosis, and length of treatment, with all *p*-values > 0.05 as shown in Table 3.

**Table 3:** Associations between sociodemographic and cancer characteristics and telehealth usability

Variable	Category	Telehealth usability questionnaire		
		*N*	Mean ± SD	*p*-value
Age in years	29–44 Years	8	5.52 ± 1.06	0.220
	45 + Years	11	4.94 ± 0.94	
Sex	Male	5	5.96 ± 0.78	0.140
	Female	29	5.05 ± 1.30	
Education level	Post Secondary training and below	4	5.19 ± 0.87	0.989
	Third Level and Above	31	5.18 ± 1.30	
Employment status	Working full time	12	5.24 ± 1.52	0.835
	Others	23	5.15 ± 1.12	
Residence	Urban	18	4.94 ± 1.37	0.174
	Rural	16	5.53 ± 1.06	
Time since diagnosis	Less than 2 Years	4	5.75 ± 0.76	0.570
	2–5 years	23	5.05 ± 1.39	
	6 and above Years	4	5.38 ± 0.32	
Time since completing primary treatment	Less than 2 Years	17	5.21 ± 1.04	0.639
	2–5 years	6	4.63 ± 2.11	
	6 and above Years	3	5.23 ± 0.17	

### Motivations to participate in the online programme, support received and useful aspects

Analysis of participant responses revealed several key themes regarding their motivations to enroll in the programme, perceived programme benefits, and supports received. Primarily, participants were motivated by a desire for peer connection and psychosocial support, valuing the opportunity to interact with others who shared similar experiences, share their own stories, and learn from one another. The programme's creation of a safe space for open communication and sharing fostered a sense of community among participants, which they greatly appreciated. Access to practical support tools, including technical assistance, end-of-programme resources, and family and caregiver support, was also highly valued. Additionally, the programme's accessibility, particularly its low or no-cost nature, was noted as an important factor. These themes and their illustrative quotes are summarized in Table 4.

**Table 4:** Participant motivations, support received and usefulness of programme aspects

Area	Category/theme	Description	*N*	Illustrative quotes
Motivations	Peer connection and interaction	Opportunity to interact with others who have had similar experiences to them. Participants valued the chance to communicate, connect, and speak with other persons impacted by cancer, as well as the sense of understanding and shared experience that comes from this interaction	19	*"To connect with others who would understand my thought, worries and feelings surrounding cancer."* *"To communicate with other people who have gone through a similar experience”*
	Seeking psychosocial support	Seeking support to address psychosocial aspects of cancer diagnosis such as fear of progression and recurrence and general psychological support such as anxiety	21	*“Needed support struggling with anxiety”* *“To get the tools to help myself heal and to meet other people in my situation."* *‘Expert Psychosocial support and discussion with other cancer survivors’*
	Moving on	This could involve, regaining confidence, or transitioning from a patient to a 'survivor' mentality and returning to work	12	*"To try to move on psychologically."* *“To move on and gain confidence"* *“To process the diagnosis before returning to work’*
	Comparison and validation	The opportunity to compare their progress and experiences with those of others	2	*“To communicate with people who went through a similar experience to me and to gauge where I was in my recovery in comparison to others."* *“I wanted to gauge where I was in my recovery in comparison to others who have experienced a similar illness."*
	Covid- 19 Pandemic	Covid-19 Restrictions reduced in person engagements, so this was only option available	3	*“Due to Covid, this was the only online support service available."* *“I didn't get a chance to interact much with other individual patients during my treatment period due to Covid restrictions”*
Programme aspects	Peer sharing and learning	Interacting with others who had similar experiences. In addition, they valued the chance to share their experiences and learn from others. They found it helpful to receive advice from people who had experienced a similar illness and to pass on the learnings they had gained along their treatment or illness journey	20	*“Being able to interact with other individuals who have been through a similar experience”* *“To be able to share some learnings I had gained along my treatment/ illness journey."* *“To receive advice from people who have experienced a similar illness”*
	Safe spaces and facilitation	A safe space to express themselves freely. Additionally, they felt that excellent facilitation skills aided in promoting the safe spaces	11	*“Being able to communicate freely in a safe space”* *“Openness able to discuss diagnosis and treatment. Forum to share experiences”*
	Smaller Group Interactions or Discussions (Break out rooms)	Smaller group interactions or discussions, such as ‘break out rooms'	6	*“Breakout groups where we got to chat."* *"Interacting with the other participants in break out rooms."* *‘Making a group agreement to commit to individual goals set every week’*
	Sense of community	Feeling part of a group of individuals who had similar experiences	3	*“Sense of community with fellow survivors* *"The course leader was fantastic, there was a sense of community we still talk in our group."*
Support Received	Technical support	Guidance on how to navigate the programme and the sessions materials. These instructions were provided through various means, such as email supports, manuals, videos, or in-session demonstrations. This also included updates, notifications, reminders, or resources related to the programme	7	*“Talking me through signing into meetings step by step’* *‘Regular emails to share link to online session. Emails with documentation suitable to recovery’* *‘Phone support from the center’*
	End-of-Programme Package/Handouts	A take home handout/ package received at the end of the programme. This package contained summaries, resources, certificates, or other materials that wrap up the programme or support post-programme progress	6	*“We were provided with a book about living with long term health conditions. This book complimented the course and has been something I have referred back to after the course’’*
	Family and caregiver support	Support from spouses, family members included support to navigate the programme and/or help in responsibilities such as childcare duties	3	*‘Support from my husband so I could attend’* *‘Childminding ‘* *‘Childcare from my partner.’*
	Peer support	The term 'group' was common, suggesting that group-related support (which could include group discussions via WhatsApp, group activities, etc.)	3	*"Peer support. Making a group agreement to commit to the individual goals set each week."* *“A group what’s app, time to speak within the group’’*
	Low cost/No cost	Offering the programme free of charge	1	*‘The programme was free, so no financial support was required*

## Discussion

This study provides preliminary evidence on the usability, effectiveness, and participant experiences of the CH-delivered CTS programme in supporting psychosocial wellbeing and QoL of people living with and beyond cancer in Ireland. Findings suggest high CH usability and satisfaction, with participants finding the technology to access the programme easy to use, effective, and satisfactory. The usability scores, as measured by the TUQ [18] were high across all the categories, and this did not differ significantly across sociodemographic characteristics or cancer history. This finding is consistent with other studies that have reported high usability scores for CH systems in cancer care [48, 49]. Notably, these studies also reported a correlation between high usability and higher education and socioeconomic status, suggesting that PLWBC with greater educational attainment and financial resources, which perhaps enables them to afford devices and technologies to engage in CH, may benefit more from such CH-delivered programmes. Further, higher education and income are linked to greater digital health literacy [48] and higher CH uptake. This trend was evident in our study, where the sample was relatively highly educated. This suggests that the uptake of the CH technologies continue to affected by literacy skills, reflecting a persistent digital divide among cancer populations [50] [14]. If not addressed, this divide risks exacerbating health inequities, as healthcare digitisation continues to grow in Ireland and globally [14].

Notwithstanding potential concerns surrounding the digital divide, the ease of use and effectiveness in communication experienced in this study were particularly notable, reinforcing the importance of user-friendly interfaces in enhancing CH experiences [16, 49]. Conversely, the TUQ reliability scale received the lowest average score, suggesting that there may be concerns or perhaps areas of improvement related to the CH’s reliability and error handling in this context. CTS is delivered via video conferencing technologies, particularly Zoom, and participants can engage using various devices such as tablets, computers or smartphones, and this may explain the variability noted in error handling. While our study did not examine the devices used or the network suitability, overall, the telehealth delivery received a positive reception, suggesting a favourable rating of CH systems by the majoritys. It was also notable that technical support was provided by the centres to support those who may have encountered difficulties with the telehealth systems.

In in the present study, participants' motivations for engaging with the CTS programme included seeking peer support, psychosocial assistance, and practical tools for managing their health. Participants endorsed self-management and mental health and wellbeing as among the most useful components, with family and peer support experienced as the least satisfactory element, despite it being the most important themes. This is not surprising considering that, while participants appreciated the practical tools like self-management, upon which CTS programme is structured [34, 35], they also formed peer connection and sharing in the process. These motivations align with existing literature identifying social support and self-management as key drivers for CH interventions [51, 52], but also as among the top unmet supportive care needs for PLWBC overall [53]. The impact of COVID-19 as a motivator underscores the pandemic's role in accelerating CH adoption, a trend observed globally [11]. However, the variable perception of CH equivalence to in-person visits noted in this study highlights an area for improvement. While CH offers numerous benefits, there are still challenges in emulating the nuanced interactions of face-to-face interactions. This finding echoes other research suggesting that while CH may provide a feasible alternative for many aspects of care, certain elements of in-person visits remain unmatched [13, 51, 54]. This has been commonly termed as the lack of ‘personal touch’ in telehealth delivery.

Further, convenience offered by CH was also noted as a motivator. CH eliminates the need for travel and allows patients to engage with services and supports from their homes. This may be particularly important for PLWBC who may have caring responsibilities or who live relatively far from healthcare facilities that may be poorly served by public transport services [55], such as those in rural areas as reflected in this sample where almost half of respondents resided. In addition to motivations for participation in the programme, the supports participants received, such as technical assistance from the centres, were crucial for participant engagement and success. In CH-delivered programmes, technical supports could be amplified, specifically with respect to error handling which participants identified as a concern. Family and caregiver support and help in responsibilities such as in childminding also played a role, suggesting the need for comprehensive approaches that consider the broader social context of cancer survivorship [56]. Moreover, Darly et al.’s review reported that CH has a beneficial impact on PLWBC and their family and caregivers, extending beyond the intended health-related outcomes. One such benefit is the extended family bonding time [57]. The support is also useful in circumstances where the patient has limited technological skills, necessitating assistance from family members or caregivers. Thus, future CH delivered cancer survivorship programmes need to go beyond the patients, to families and caregivers.

Nearly all the participants completed the prescribed CTS sessions, highlighting its high acceptability and engagement. This was further evidenced by the high perceived usefulness score, an important predictor of engagement in CH [18, 58]. A web based CTS feasibility study conducted in the US also reported high acceptability, with over 95% of participants expressing satisfaction with the programme content [39]. Similar feedback was received from initial programme beneficiaries in Ireland [38] and the US [39]. This suggests that irrespective of the mode of delivery, the programme remains very attractive to those affected by cancer. The sustained engagement during the pandemic when this study was conducted and afterwards underscores CH’s potential to not only offer continuity of care, but also compliment in-person care [11].

## Implications for practice and policy

The high satisfaction and usability ratings for the online CTS programme suggest that CH technologies can effectively complement in-person support in survivorship care. These findings are important for policymakers and particularly the NCCP as it aims to enhance cancer survivorship services, amidst the rising number of PLWBC. The integration of CH technologies into routine care can increase accessibility, particularly for those in rural and underserved areas or for those with mobility issues, aligning with Ireland's digital health initiatives [30], but also with the global strategy on digital health [59]. To maximize the benefits of CH, continuous improvements in technology and support systems are essential. Enhancing the equivalence of CH to in-person visits through better video quality; error handling and more interactive features could further improve user satisfaction.

Moreover, while our findings align with studies such as Layfield et al*.* [60] and Kvedar et al*.* [42] which demonstrated CH’s efficacy in improving health outcomes, the focus on the Irish context provides unique insights into the local applicability of CH-delivered interventions. Notably, this is the first study to evaluate the usability and utility of CH-delivered CTS programme for PLWBC in Ireland. Future research should examine strategies to overcome noted challenges and barriers, ensuring that CH delivered interventions can be effectively integrated into routine cancer survivorship programmes.

## Study limitations

The small sample size may limit the generalizability of the findings. Furthermore, participation and engagement in the CH-delivered programmes were potentially influenced by the COVID-19 pandemic, underscoring the importance of ongoing programme evaluation. The brevity of the open-text responses suggests the need for more comprehensive qualitative approaches to gain a deeper understanding of the full scope of patient experiences. Additionally, the study focused on PLWBC who completed the CH-delivered programme only, highlighting the need for future comparative studies to compare outcomes with in-person delivery.

## Conclusions

The findings of this study demonstrate that the CTS programme, delivered through CH, is feasible, acceptable and helpful in supporting PLWBC in Ireland. The high usability and positive rating on supporting psychological well-being, QoL and self-management reflect the programme's potential in leveraging CH technologies to enhance survivorship care.

This work was presented as an oral presentation at the IACR 60th Anniversary Satellite Meeting, February 2024 (Dublin, Ireland).

## Data Availability

The data that support the findings of this study are available on request from the corresponding author.
